# Substance use and self-harm: a cross-sectional study of the prevalence, correlates and patterns of medical service utilisation among patients admitted to a South African hospital

**DOI:** 10.1186/s12913-018-2963-7

**Published:** 2018-03-06

**Authors:** Elsie Breet, Jason Bantjes, Ian Lewis

**Affiliations:** 1 Department of Psychology, Stellenbosch University, Private Bag X1, Matieland, Stellenbosch, Cape Town, 7602 South Africa; 2 Department of Psychiatry and Mental Health, University of Cape Town, Cape Town, South Africa

**Keywords:** Substance use, Suicidal behaviour, Self-harm, Medical service utilisation, Acute use of substances

## Abstract

**Background:**

Substance use is a potentially modifiable risk factor for suicidal behaviour. Little is known about the epidemiology of substance use among self-harm patients in South Africa. This study set out to collect epidemiological data about the prevalence, correlates, and patterns of medical service utilisation among self-harm patients who used substances at the time of self-injury.

**Methods:**

Data from 238 consecutive self-harm patients treated at an urban hospital in South Africa were analysed using bivariate and multivariate statistics.

**Results:**

Approximately 20% of patients reported substance use at the time of self-harm. When compared to other self-harm patients, higher rates of patients who had used substances: had depressed levels of consciousness on admission; utilised more medical resources and required longer hospital admissions; cited relationship difficulties and financial concerns as reasons for their self-harm; reported a previous episode of self-harm; and intended to die as a result of their injuries. Although the observed differences were not statistically significant (*p* > 0.05), the proportional differences were congruent with international literature.

**Conclusion:**

Acute use of substances among self-harm patients warrants more focused research and clinical attention particularly in the context of reducing utilisation of scarce medical resources.

**Electronic supplementary material:**

The online version of this article (10.1186/s12913-018-2963-7) contains supplementary material, which is available to authorized users.

## Background

Substance use is a recognised risk factor for self-harm (i.e., intentional, non-habitual self-injury with or without intent to die) and completed suicide [1]. Extensive literature documents the relationship between alcohol use and self-harm [2, 3]. A comparatively smaller body of literature describes associations between self-harm and the use of cannabis [4], heroin [5], methamphetamines [6] and cocaine [7]. Literature in this area comes predominantly from high-income Western countries. Research from low and middle-income countries is relatively scant despite the fact that 75.5% of all suicides occur in these countries [8]. To date no studies in South Africa (SA) document the prevalence and correlates of substance use among persons who engage in self-harm, although there is some evidence to suggest that completed suicides in the country are associated with substance use [9]. This study documents the prevalence and correlates of acute use of substances (AUS) (i.e., substance use during or shortly before engaging in self-injurious behaviour) [10] among patients treated at an urban hospital in SA.

### Acute use of substances and self-harm in high-income countries

An abundance of literature from high-income countries report on the association between AUS and self-harm. The findings from cross-sectional studies show that AUS is associated with self-harm among males [11], and younger adults [1]. Moreover, evidence suggests that AUS (in particular the quantity of substances used and the time between substance use and self-harm) is associated with methods of self-harm that entail damage to body tissues such as hanging, cutting, burning, and gun shots [12]. High levels of substance use during social events are associated with self-harm, independent of the level of suicidal intent, as a result of disinhibition, increased impulsivity, impaired judgement, increased feelings of depression or hopelessness, and the urge to escape a situation or to change the behaviour of someone else [13, 14]. In cases with higher levels of intent to die and premeditation, AUS may facilitate self-harm by easing the distress of engaging in self-harm [15].

Substance use by self-harm patients may influence their medical management, the treatment they receive and the clinical decisions of medical staff. Ries and colleagues reported that self-harm patients who had used substances at the time of injury were discharged sooner than patients whose behaviour was not perceived to be related to substance use [16]. Clinicians believe that AUS related self-harm is linked to lower levels of premeditation (i.e., is more impulsive) and lower levels of suicidal intent [15]. This belief together with aggressive or uncontrolled behaviour upon arrival at the emergency department, seems to lead to self-harm patients who had been using substances receiving less intensive medical care and being discharged sooner than patients who did not have a substance-related risk for suicidal behaviour [17].

### Substance use and suicidal behaviour in South Africa

Alcohol is the most common substance used in SA [18]. Widespread unrecorded alcohol production (e.g., home-brewing of beer) and illegal selling of alcohol, make it difficult to accurately estimate alcohol consumption in SA [19]. Yet, available prevalence rates of lifetime alcohol use in the Western Cape range from 39% to 64%, while rates of risky drinking range from 9% to 34% [20]. Data on illicit drug use in SA is limited. Cannabis is the most common drug used (approximately 2% of the population), followed by cocaine (0.3%), sedatives (0.3%), amphetamines (0.2%), inhalants, hallucinogens, and opiates (0.1% each) [21]. However, these rates are thought to be underestimated with a report from the International Narcotics Board suggesting that up to 15% of the country’s population regularly use some form of illicit drug [22].

Approximately 11% of non-natural deaths in SA are suicide-related [23] and an estimated 40% of suspected deaths by suicide test positive for assays of blood alcohol [9]. Nationally representative data from the South African Stress and Health study suggest that substance use disorder increased the risk for suicide attempts (OR = 4.1), more than any other common mental disorder [24].

Better understanding the relationship between AUS and self-harm in SA may help identify strategies to reduce the morbidity and mortality associated with self-harm and to lessen the burden placed on the health care system; a system that is already under-resourced and overburdened [25, 26]. The aim of this study was to collect epidemiological data about the prevalence and correlates of AUS among self-harm patients seeking treatment in an urban hospital in the Western Cape Province of SA (*hereafter referred to as the hospital*). A secondary aim was to describe the pattern of medical service utilisation among this group of self-harm patients.

## Methods

### Data collection procedures

This study consisted of a point prevalence sample from a small population by collecting data from all self-harm patients who presented for treatment at the hospital between 16 June 2014 and 29 March 2015. During this time there were 270 consecutive presentations of self-harm, of which 238 were eligible for inclusion. Cases were excluded if: the files were missing or there was not sufficient information available in the patient file (17 patients); the patient had already been included in the sample on a prior presentation to the hospital during the period of data collection (9 patients); patients left the hospital before data was captured (1 patient); or patients died as a result of their injuries (5 patients). Sample size calculations were based on 15 patients per predictor variable in each model of logistic regression [27].

### Definition of self-harm

In this study self-harm is defined as intentional self-injury or self-poisoning with non-fatal outcome, regardless of the degree of intent to die, which is deliberate and is non-habitual [1, 8, 28]. The term self-harm, as we have defined it, is synonymous with the term ‘deliberate self-harm’ and includes suicide attempts but does not include habitual or repetitive self-injury. We did not confine our study to suicide attempts because we could not reliably determine intent to die in order to differentiate suicide attempts from other forms of deliberate self-harm. This inclusion of cases of self-harm regardless of the level of intent is consistent with the approach adopted in other studies [29] and with the WHO’s inclusion of self-harm as a component of non-fatal suicidal behaviour [8]. Within our definition of self-harm, substance use would not be considered a form of self-harm since this behaviour is both habitual and the use of substances is not primarily motivated by a conscious desire to inflict bodily harm.

### Measures

The following data was collected:

#### Demographic information

Patient’s age, gender, ethnicity, home language, relationship status, number of dependents, level of education, and employment status. It is worth noting that the hospital only treats patients 13 years and older. Socio-economic status (SES) was also recorded as low to moderate SES (ZAR0 to ZAR76 800) and high SES (ZAR76 801 to ZAR2 547,601) based on annual family income.

#### Substance use

Self-reports of substance use at the time of self-harm, and type of substance(s) used. Rates of substance use may be limited by underreporting when relying on self-report measures. However, in SA self-report measures for harmful alcohol use and drug-related problems showed some agreement when compared to the use of biomarkers to determine substance use [30]. Studies that compare self-report measures with more objective measures of illicit use of other substances are scant.

#### Clinical features of self-harm

Method(s) of self-harm; medical intervention(s) received; psychiatric assessment conducted; level of admission required (i.e., treated and discharged, or admitted to the ICU, high care, medical / surgical ward, or emergency psychiatric unit), and length of stay in the hospital.

#### Level of consciousness on admission

The Glasgow Coma Scale (GCS) was used to measure the level of responsiveness to stimuli (i.e., level of consciousness) on arrival at the hospital. This study considered a score of 13 to 15 to indicate no or minimal depression in level of consciousness (LOC), a score of 9 to 12 indicated moderately depressed LOC, and a score of 8 or less indicated significantly depressed LOC.

#### Level of suicidal intent

The 12-item Pierce Suicidal Intent Scale (PSIS) was used to measure suicidal intent among patients [31]. The PSIS scores range from zero to 25, where scores between zero and three indicates low suicidal intent, scores between four and 11 indicates moderate suicidal intent, and scores higher than 11 indicates severe suicidal intent. In this study, only three AUS patients reported low suicidal intent. The small number of patients makes meaningful analysis difficult. Therefore, two categories were created for the analysis consisting of low to moderate suicidal intent (i.e., scores between zero and 11) and high suicidal intent (i.e., scores higher than 11).

#### Details of self-harm

Patient’s self-report of the stated intention for self-harm, reasons for engaging in the behaviour, history of a previous episode of self-harm, and whether the incident was impulsive (as opposed to planned).

The data was collected from patient records. The recording of this data is part of the routine clerking of all self-harm patients in the hospital. Quality checks for possible errors and missing data were done throughout data collection.

### Statistical analyses

Data were analysed using SPSS v.18. Following descriptive analyses, bivariate analyses of the association between AUS and self-harm were performed using chi-square statistics or Fisher’s exact test for categorical variables. Odds ratios (OR) and 95% confidence intervals (CI) were calculated for variables with significant *p*-values. The Mann-Whitney test was used for between-group analyses of continuous variables with nonnormal distributions. Logistic regression analysis was used to explore the relationship between AUS and gender, SES, having dependents, suicidal intent, history of previous self-harm, LOC on admission, medical intervention required, level of suicidal intent, whether a psychiatric assessment was received, hospital admission required, and whether the self-harm was impulsive as opposed to planned. Statistical significance was set at *p* < 0.05.

### Ethical approval

Ethical approval for this study was granted by the Health Sciences Research Ethics Committee: at Stellenbosch University (reference number: N13/05/074) and University of Cape Town (reference number: 645/2013). Written permission was granted by the hospital before patient records were accessed. Information collected from patient records were saved on a password protected computer where each patient was assigned a unique number to protect patient confidentiality.

## Results

### Demographic characteristics of sample

The sample consisted of 238 self-harm patients, of which 20.2% reported substance use at the time of self-injury (i.e., AUS). The demographic characteristics of the sub-group of AUS patients and other self-harm patients with no AUS is shown in Table 1. The mean age of AUS patients was 32.9 (SD = 11.8) years, while the mean age of other self-harm patients was 31.2 (SD = 14.3) years. The sub-group of AUS patients were predominantly male (52.1%), black (37.5%), not in a relationship (85.4%), unemployed (72.9%), and were of low- to moderate SES (43.8%).

**Table 1:** Description and comparison of sample demographic characteristics, by acute use of substances

Variable	Yes ^a^*n* = 48 (%)	No ^b^*n* = 190 (%)	χ^2^	df	*p* -value	OR (CI)
Gender						
Male	25 (52.1)	71 (37.4)	3.45	1	0.063	
Female	23 (47.9)	119 (62.6)				
Mean (SD) Age (years)	32.9 (11.8)	31.2 (14.3)				
^c^Race			7.93	4	0.094	
Black	18 (37.5)	64 (33.7)				
Asian/Moslem	1 (2.1)	7 (3.7)				
^d^Coloured	15 (31.3)	88 (46.3)				
White	12 (25.0)	21 (11.1)				
Not known	2 (4.2)	10 (5.3)				
Relationship status				–	–	–
Married/In a relationship	7 (14.6)	39 (20.5)				
Not in a relationship	41 (85.4)	150 (78.9)				
Not Known	0	1 (0.5)				
Have dependents			5.95	1	0.015	2.59 (1.12–6.12)
No dependents/pregnant	39 (81.3)	119 (62.6)				
Dependents	9 (18.8)	71 (37.4)				
^e^Completed level of education			5.92	2	0.052	
Primary school	24 (50.0)	76 (40.0)				
Secondary school	13 (27.1)	87 (45.8)				
Tertiary school (Undergraduate or postgraduate)	11 (22.9)	27 (14.2)				
^f^Employment status			–	–	–	
Employed	13 (27.1)	44 (23.2)				
Unemployed (unemployed, scholar, student)	35 (72.9)	141 (74.2)				
Not known	0	5 (2.6)				
^g^SES			5.23	2	0.073	
Low to moderate SES (ZAR0 to ZAR76 800)	21 (43.8)	110 (57.9)				
High SES (ZAR76 801 to ZAR2 547,601)	19 (39.6)	66 (34.7)				
Not known	8 (16.7)	14 (7.4)				

Total sample = 238. OR = Odds Ratio; CI = confidence intervals. Chi-square statistics were calculated for categorical variables: gender, race, having dependents or no dependents, completed level of education, and socio-economic status (SES). Mann-Whitney U test was used for between-group analyses of continuous variables with nonnormal distribution: Mean age (years)

^a^*n* = 48; ^b^*n* = 190; ^c^Race = the term race may be offensive in some countries, however this is an official term used in South Africa. ^d^Coloured = the term coloured may be offensive in some countries, however this is an official term used in South Africa. ^e^Primary school = 1st grade to 7th grade in the United States; Secondary school = 8th grade to 12th grade/Senior in the United States; Tertiary school = any Diploma or University degree after completing Grade 12. ^f^Employment status = 6 participants who indicated that they were retired were included in the employed category as they qualify to receive old age pension from the state worth ZAR1420 per month. ^g^ZAR15.72 = 1 US dollar

AUS self-harm patients were approximately 2.6 times more likely not to have dependents [x^2^ = 5.95 (df = 1), *p* = 0.015, OR = 2.59, 95% CI 1.122–6.119], when compared to those who had not used any substances at the time of their injuries. In the logistic regression analysis, demographic characteristics (i.e., gender, SES, and having dependents) did not predict AUS when controlling for the other variables in the model (see Additional file 1: Table S1).

### Range of substances used

The range of substances used by self-harm patients is provided in Table 2. Alcohol (73%) was the most commonly used substance, while methamphetamine (10.4%), cannabis (6.25%), cocaine (6.25%), and heroin (4.17%) use was also reported. Only one patient reported using opioids. Multiple substance use at the time of the self-injury was reported by 19% of the sample.

**Table 2:** Description and comparison of clinical features by acute use of substances

Variable	Yes ^a^*n* = 48 (%)	No ^b^*n* = 190 (%)		χ^2^	df	*p-*value
^c^Acute substance used						
Alcohol	35 (72.9)					
Cannabis	3 (6.25)					
MDMA (Ecstasy)	1 (2.08)					
Methaqualone (Mandrax)	2 (4.17)					
Cocaine	3 (6.25)					
Methamphetamine (Tik)	5 (10.4)					
Heroin	2 (4.17)					
Opiates	1 (2.08)					
Multiple substance use						
Yes	9 (18.8)					
No	39 (81.3)					
Method of self-harm						
Self-poison	37 (77.1)	154 (81.1)		0.381	1	0.537
Prescription medication	27 (56.3)	118 (62.1)		0.552	1	0.458
Non-prescription medication	17 (35.4)	57 (30.0)		0.525	1	0.469
Ingestion or inhalation of poison	3 (6.25)	22 (11.6)		1.16	1	0.429
Damage body tissue	8 (16.7)	26 (13.7)		0.278	1	0.598
Laceration	4 (8.33)	18 (9.47)		0.040	1	0.769
Hanging	3 (6.25)	13 (6.84)		0.021	1	1.00
Asphyxiation	1 (2.08)	1 (0.53)		1.12	1	0.291
Immolation	1 (2.08)	0		–	–	–
Jumped off a height	2 (4.17)	1 (0.53)		0.332	1	0.564
Jumped in front of a train	1 (2.08)	2 (1.05)		0.327	1	0.493
Mixed method (i.e. self-poison and damage to body tissue)	3 (6.3)	8 (4.2)		0.290	1	0.703
Not known	0	2 (1.1)		–	–	–
^d^Glasgow Coma Scale (Level Of Consciousness)				0.099	2	0.095
Minimal depression in LOC	40 (83.3)	161 (84.7)				
Moderately depressed LOC	3 (6.3)	12 (6.3)				
Significantly depressed LOC	5 (10.4)	17 (8.9)				
Received medical intervention				0.269	1	0.604
Yes	32 (66.7)	119 (62.6)				
No	16 (33.3)	71 (37.4)				
^e^Pierce Suicide Intent Scale (PSIS)				0.079	2	0.961
Low to moderate suicide intent	17 (35.4)	69 (36.3)				
High suicide intent	8 (16.7)	34 (17.9)				
Not known	23 (47.9)	87 (45.8)				
Received a psychiatric assessment				0.031	1	0.859
Yes	34 (70.8)	137 (72.1)				
No	14 (29.2)	53 (27.9)				

Total sample = 238. Chi-square statistics were calculated for categorical variables: level of consciousness, medical intervention received, level of suicidal intent, and whether or not a psychiatric assessment was received

^a^*n* = 48; ^b^*n* = 190; ^c^Description of type of, and single or multiple acute substance use; ^d^No or minimal depression in level of consciousness = a score of 13 to 15 on the Glasgow Coma scale; moderately depressed level of consciousness = a score of 9 to 12 on the Glasgow Coma scale; significantly depressed level of consciousness = a score of 8 or less on the Glasgow Coma scale. ^e^Low to moderate suicide intent = a PSIS of 11 or lower; high suicide intent = PSIS score of 12 or more

### Methods of self-harm

The methods of self-harm are provided in Table 2. Self-poisoning was the most common method of self-harm among AUS patients (77.1%). Among patients who reported self-poisoning, prescription medication was the most common method used among AUS patients (56.3%). There was no statistically significant association between status of AUS (i.e., AUS patients and other self-harm patients) and the methods of self-harm employed. That is, AUS patients and other self-harm patients equally reported the use of different methods of self-harm (Table 2). In regression models, AUS did not predict whether patients used self-poisoning or damage to bodily tissue (see Additional file 1: Table S1).

### Level of consciousness on admission and medical interventions required

A greater proportion of AUS, compared to other self-harm patients, had moderate to severe depressed LOCs on admission (16.7% vs. 15.2%), and required medical intervention (66.7% vs. 62.6%). There was no statistically significant association between status of AUS (i.e., AUS patients or other self-harm patients) and LOC (Table 2). In the regression analysis, AUS did not predict patients’ LOC, or whether a medical intervention was required (see Additional file 1: Table S1).

### Suicidal intent

A slightly smaller proportion of AUS, compared to other self-harm patients, were assessed as having high levels of suicidal intent (16.7% vs. 17.9%), and were referred for psychiatric assessment (70.8% vs. 72.1%). There was no statistically significant association between status of AUS (i.e., AUS patients or other self-harm patients) and levels of suicidal intent (Table 2). AUS did not predict suicidal intent (see Additional file 1: Table S1).

### Level of admission and length of hospital stay

Level of hospital admission required by self-harm patients and their length of stay in hospital, are provided in Additional file 1: Table S2. Compared to other self-harm patients, AUS self-harm patients spent more time in short stay medical units [M = 3.44 days (SD = 2.06) vs. M = 2.64 days (SD = 1.54)], long stay medical or surgical wards [M = 25 days (SD = 34.1) vs. M = 15.1 days (SD = 22.4)], ICU or high care [M = 6.25 days (SD = 3.27) vs. M = 4.62 days (SD = 3.69)], and in an emergency psychiatric unit [M = 6.80 days (SD = 5.89) vs. M = 6.46 days (SD = 5.54)], although these differences were not statistically significant. AUS did not significantly predict whether patients received a psychiatric assessment, whether patients were treated in the emergency department and discharged, or admitted to a long stay medical ward (see Additional file 1: Table S1). Likewise, AUS did not predict whether patients were admitted to the ICU or a high care medical unit (see Additional file 1: Table S1).

### Stated intention

The most common intentions reported by AUS patients were to escape a situation (22.9%) and to communicate something (e.g., distress) (27.1%) (Table 3). There was no statistically significant association between status of AUS (i.e., AUS patients or other self-harm patients) and stated intension. .

**Table 3:** Comparison of stated intention and reason, previous attempts, and impulsivity, by acute use of substances

Variable	Yes ^a^*n* = 48 (%)	No ^b^*n* = 190 (%)	χ^2^	df	*p*-value	OR (CI)
Stated Intention						
To regulate the behaviour of someone else	6 (12.5)	49 (25.8)	3.81	1	0.051	
To regulate emotional state	3 (6.3)	22 (11.6)	1.16	1	0.429	
To escape a situation	11 (22.9)	37 (19.5)	0.282	1	0.595	
To communicate something (e.g. distress)	13 (27.1)	69 (36.3)	1.45	1	0.229	
Mistake	5 (10.4)	9 (4.74)	2.23	1	0.135	
Chronic physical pain/illness	1 (2.1)	2 (1.05)	0.327	1	0.493	
Not known	5 (10.4)	10 (5.26)	1.72	1	0.192	
Suicidal self-injury (i.e., ‘to die’ as one of their reasons)						
To die	21 (43.8)	60 (31.6)	2.53	1	0.126	
Other	22 (45.8)	118 (62.1)	4.19	1	0.041	
Not known	5 (10.4)	12 (6.32)	0.972	1	0.324	
Stated Reason						
Financial concerns	13 (27.1)	34 (17.9)	2.04	1	0.153	
Friendship/Marital/romantic relationship issues	16 (33.3)	58 (30.5)	0.141	1	0.707	
Family conflict	11 (22.9)	76 (40.0)	4.82	1	0.028	2.24 (1.02–5.00)
Social issues (i.e., isolation, friendship problems, legal issues)	2 (4.2)	10 (5.26)	31.3	1	0.000	24.7 (4.79–170.9)
Medical illness	6 (12.5)	12 (6.32)	2.10	1	0.148	
Psychiatric illness	8 (16.7)	28 (14.7)	0.111	1	0.739	
Bereavement	3 (6.3)	7 (3.68)	0.627	1	0.426	
Academic concerns	2 (4.2)	13 (6.84)	0.464	1	0.742	
Unplanned pregnancy	0	3 (1.58)				
Not known	7 (14.6)	22 (11.6)	0.323	1	0.570	
Previous attempt of self-harm					0.340	
Previous attempt	22 (45.8)	67 (35.3)				
No previous attempt	11 (22.9)	58 (30.5)				
Not known	15 (31.3)	65 (34.2)				
Impulsive act					0.382	
Yes	9 (18.8)	47 (24.7)				
No	39 (79.2)	143 (75.3)				

Total sample = 238. Chi-square statistics were calculated for categorical variables: stated intention, stated reason, previous attempt of self-harm, and impulsive act

^a^*n* = 48; ^b^*n* = 190

### Suicidal self-injury

A greater proportion of AUS, when compared to other self-harm patients, stated that their intention was ‘to die’ (43.8% vs. 31.8%) There was no statistically significant association between status of AUS (i.e., AUS patients or other self-harm patients) and stated intension to die (Table 3). AUS did not predict whether patients reported that they intended to die (see Additional file 1: Table S1).

### Stated reasons for self-harm

The most common reasons given for self-harm among AUS patients were relationship (friendship, marital, or romantic) issues (33.3%), financial concerns (27.1%), and family conflict (22.9%) (Table 3). There was no statistically significant association between status of AUS (i.e., AUS patients or other self-harm patients) and stated reason for self-harm (see Additional file 1: Table S1).

### History of self-harm

Approximately half (45.8%) of AUS patients reported one or more previous episodes of self-harm, while 35.3% of other self-harm patients reported a previous episode of self-harm. There was no statistically significant association between status of AUS (i.e., AUS patients or other self-harm patients) and history of self-harm (Table 3).

### Impulsive self-harm

A smaller proportion of AUS patients (18.8%) reported that their self-harm was impulsive compared to other self-harm patients (24.7%). There was no statistically significant association between status of AUS (i.e., AUS patients or other self-harm patients) and whether or not the self-harm was impulsive (Table 3). In the logistic regression, AUS did not predict whether the self-harm was impulsive (see Additional file 1: Table S1).

## Discussion

This study represents a small but important first step towards better understanding the relationship between AUS and self-harm in SA. In our sample, one in five self-harm patients presenting for treatment at an urban hospital reported that they had used substances at the time of their self-harm.

Compared to other self-harm patients in the sample, a greater proportion of patients who had used substances at the time of their self-harm had a depressed level of consciousness on admission, required a medical intervention, were admitted to an ICU or high care unit, had longer hospital stays than other self-harm patients, and reported that they intended to die as a result of their injuries. These associations calculated using the Chi-Square statistical test were not statistically significant (*p* > 0.05), though the overall direction of the findings were in keeping with studies from high-income countries [12, 15, 32]. It is possible that given a larger sample and more accurate measures of AUS, we would have found the differences we observed to be statistically significant. This increased level of medical service utilisation among self-harm patients who had used substances at the time of their injuries highlights the economic importance of addressing this health problem, particularly in the light of scarce medical resources in SA [25, 26].

It is noteworthy that a higher proportion of self-harm patients who had used substances report a history of previous episodes of self-harm, compared to other self-harm patients. This association was not found to be significant (*p* > 0.05) when using the Chi-Square statistical test, though the overall direction of the findings is consistent with the international literature [33] and highlights the need for targeted interventions to reduce the risk of repetition among this population of patients.

The self-harm patients in this sample were all in contact with the health care system as a result of the injuries they had sustained. This contact with the health care system represents a potential opportunity for targeted interventions to address substance use problems, which seems to be important given the finding that 20% of self-harm patients had used substances at the time of their self-harm. Given that substance use is associated with risk of repetition of self-harm and eventual death by suicide [34], it seems sensible to utilise this contact with the health care system to address unhealthy patterns of substance use. It may be beneficial to routinely screen for problem substance use among self-harm patients and to offer referrals to appropriate substance use treatment centers or, where appropriate, to deliver brief targeted interventions as part of the management of self-harm at the hospital. This is particularly important in SA where there are high rates of substance use and problematic patterns of alcohol use [18, 22]. The lack of accessible, affordable and effective treatments for substance use problems in SA [35] is a significant barrier to suicide prevention.

There are some limitations to this study. As the data was collected from one hospital setting it is not appropriate to generalise the findings to other settings. Future research should replicate this study by collecting data from different hospitals across multiple settings. Some caution is necessary when interpreting the findings since this study recruited a consecutive sample, which is a type of non-random purposive sampling and as such may result in selection bias. This study did not take account of variables such as the quantity of substances consumed, the context in which substances were consumed (i.e., socially versus in isolation) and the proximity of substance use to the incident of self-harm, which are potentially important variables. The data were collected from a retrospective review of patient files that included self-reported information on demographic information, substance use, and details of self-harm that was collected during routine assessment of self-harm patients. The nature of the self-report measures used may have contributed to rates of not known data with regard to demographic information and details of self-harm. Likewise, given that patients may be reluctant to report substance use honestly [36] it is likely that the prevalence of AUS is lower than would have been found had we used more objective measures of substance use (e.g. testing blood alcohol concentration upon admission to the hospital). Furthermore, the use of more objective self-report measures for substance use could have allowed for the exploration of important aspects of substance use and self-harm that have been highlighted in the literature but were not part of the routine assessment that patients received. In this study, it would have been helpful to collect data that make a distinction between types of substance use, determined whether substance use was at a risky level, established when the individuals were exposed to the substance in proximity to self-harm, and assessed the pattern of substance use. Objective measures that would be appropriate to use in this context include but are not limited to the Alcohol, Smoking and Substance Involvement Screening Test (ASSIST), Alcohol Use Disorder Identification Test (AUDIT), and the Drug Use Disorder Identification Test (DUDIT). With regard to self-harm, the Columbia-Suicide Severity Rating Scale or the Mini International Neuropsychiatric Interview 6 (Module B) could provide insight into the past or present suicidal ideation, and details of past suicidal behaviour. Future research should also assess substance use patterns of behaviour and previous AUS without an act of self-harm. This study did not control for the influence of confounders such as: substance use disorder and comorbid psychiatric disorders and the context in which the substances were consumed. Future studies may shed light on the extent to which a history of substance use among this sub-group of patients may have played a role in causing the relationship and financial problems that precipitated their self-harm. The findings are an important first step in drawing attention to the prevalence and correlates of acute substance use among self-harm patients in SA.

## Conclusion

Substance use is a potentially modifiable risk factor for suicidal behaviour, yet knowledge on the epidemiology of substance use among self-harm patients in SA is scant. This study provides epidemiological data about the prevalence, correlates, and patterns of medical service utilisation among self-harm patients who reported substance use at the time of their self-harm. Given that AUS among self-harm patients is a public health problem in SA, future research should seek to investigate the context in which AUS plays a role in self-harm. This would be important especially in the context of the need to reduce the utilisation of scarce medical resources in the country and address the public health problems associated with substance use.

## Additional file

Additional file 1: **Table S1.** Level of admission required and length of stay in hospital by Acute Use of Substances. **Table S2.** Binary Logistic Regression Analysis: Summary of Predictors in Each Model. (DOCX 20 kb)

## Abbreviations

AUS: Acute use of substances
CI: Confidence interval
GCS: Glasgow Coma Scale
ICU: Intensive care unit
LOC: Level of consciousness
OR: Odds ratio
PSIS: Pierce Suicidal Intent Scale
SA: South Africa
SD: Standard deviation
SES: Socio-economic status
WHO: World Health Organization
ZAR: Zuid-Afrikaanse Rand
