# Supplementary material for: Substance use and self-harm: a cross-sectional study of the prevalence, correlates and patterns of medical service utilisation among patients admitted to a South African hospital
Source: BMC Health Serv Res. 2018 Mar 6;18:157. doi: 10.1186/s12913-018-2963-7 (PMC5840832; doi:10.1186/s12913-018-2963-7)
Supplement: Supplementary file 1 — Table S1. Level of admission required and length of stay in hospital by Acute Use of Substances. Table S2. Binary Logistic Regression Analysis: Summary of Predictors in Each Model. (DOCX 20 kb) [file 12913_2018_2963_MOESM1_ESM.docx]

**Table S1** Binary Logistical Regression Analysis: Summary of Predictors in Each Model

| Model | Predictor | Outcome | B (SE) | Wald *X^2^* | *p*-value | OR | CI |  |
| --- | --- | --- | --- | --- | --- | --- | --- | --- |
| 1 | Gender | AUS | 0.694 (0.478) | 2.11 | 0.146 | 2.00 | 0.785-5.11 |  |
|  | SES | AUS | -0.494 (0.486) | 1.03 | 0.310 | 0.610 | 0.235-1.58 |  |
|  | Dependents | AUS | 0.401 (0.533) | 0.565 | 0.452 | 1.49 | 0.525-4.24 |  |
|  | Stated intention (die or other) | AUS | -0.465 (0.469) | 0.984 | 0.321 | 0.628 | 0.250-1.58 |  |
|  | Previous episode of self-harm | AUS | -0.387 (0.499) | 0.600 | 0.439 | 0.679 | 0.255-1.81 |  |
| 2 | AUS | Self-poison vs. damage to bodily tissue | -0.247 (0.444) | 0.311 | 0.577 | 0.781 | 0.327-1.86 |  |
| 3 | AUS | ^a^GSC (No or minimal depression of LOC) | -0.169 (0.539) | 0.098 | 0.754 | 0.845 | 0.294-2.43 |  |
|  |  | ^a^GCS (Moderately depression of LOC) | -0.163 (0.822) | 0.039 | 0.843 | 0.850 | 0.170-4.26 |  |
| 4 | AUS | Intervention received | -0.241 (0.347) | 0.483 | 0.487 | 0.786 | 0.398-1.55 |  |
| 5 | AUS | ^b^PSIS | 0.046 (0.447) | 0.009 | 0.923 | 1.05 | 0.411-2.67 |  |
| 6 | AUS | Assessed by a Psychiatrist | -0.013 (0.397) | 0.001 | 0.975 | 0.987 | 0.454-2.15 |  |
| 7 | AUS | ED& Discharge vs. long stay medical ward | 0.084 (0.337) | 0.063 | 0.802 | 1.09 | 0.562-2.11 |  |
| 8 | AUS | Admitted to emergency psychiatry | -0.203 (0.329) | 0.379 | 0.538 | 0.817 | 0.428-1.56 |  |
| 9 | AUS | Admitted to ICU or high care medical unit | 0.213 (0.596) | 0.128 | 0.720 | 1.24 | 0.385-3.98 |  |
| **Table S1** Binary Logistical Regression Analysis: Summary of Predictors in Each Model *(Continued)* | | | | | | | | |
| Model | Predictor | Outcome | B (SE) | Wald *X^2^* | p-value | OR | CI |  |
| 10 | AUS | Suicidal self-injury vs. non suicidal self-injury | -0.630 (0.344) | 3.356 | 0.067 | 0.533 | 0.272-1.05 |  |
| 11 | AUS | Impulsive act | 0.304 (0.412) | 0.546 | 0.460 | 1.36 | 0.605-3.04 |  |

Note: OR = odds ratio; CI = confidence intervals; AUS = acute use of substances; SES = socio-economic status.

*^a^*No or minimal depression in level of consciousness = a score of 13 to 15 on the Glasgow Coma scale; moderately depressed level of consciousness = a score of 9 to 12 on the Glasgow Coma scale; significantly depressed level of consciousness = a score of 8 or less on the Glasgow Coma scale. *^b^*Low to moderate suicide intent = a PSIS of 11 or lower; high suicide intent = PSIS score of 12 or more.

**Table S2** Level of Admission required and length of stay in hospital by Acute Use of Substances

|  | Number of patients | | χ^2^ | df | *p*-value | Total number of days spent in each ward | | Mean number of days (standard deviation) | |
| --- | --- | --- | --- | --- | --- | --- | --- | --- | --- |
|  | ^a^n(%) | ^b^n(%) |  |  |  | ^a^n | ^b^n | ^a^n | ^b^n |
| Treated in casualty and discharged | 16 (33.3) | 68 (35.8) | 0.101 | 1 | 0.750 | - | - | - | - |
| Admitted to short stay medical unit | 9 (18.8) | 55 (28.9) | 2.03 | 1 | 0.155 | 31 | 145 | 3.44 (2.06) | 2.64 (1.54) |
| ICU or high care | 4 (8.3) | 13 (6.84) | 0.128 | 1 | 0.720 | 25 | 60 | 6.25 (3.27) | 4.62 (3.69) |
| Admitted to long-stay medical or surgical ward | 6 (12.5) | 11 (5.79) | 2.60 | 1 | 0.107 | 150 | 166 | 25 (34.1) | 15.1 (22.4) |
| Admitted to emergency psychiatric unit | 20 (41.7) | 70 (36.8) | 0.379 | 1 | 0.538 | 136 | 452 | 6.80 (5.89) | 6.46 (5.54) |
| Transferred to tertiary psychiatric hospital | 5 (10.4) | 12 (6.32) | 0.972 | 1 | 0.324 | - | - | - | - |

Note: N = 238; Chi-square statistics were calculated for categorical variables: treated in casualty and discharged; admitted to short stay medical unit; ICU or high care; admitted to long-stay medical or surgical ward; admitted to emergency psychiatric unit; transferred to tertiary psychiatric hospital. Mann-Whitney U test was used for between-group analyses of continuous variables with nonnormal distribution: Mean number of days spent in each unit.

^a^n = 48 patients with acute use of substances; ^b^n = 190 of other self-harm patients.
